# Crystal structure and insights into the oligomeric state of UDP-glucose pyrophosphorylase from sugarcane

**DOI:** 10.1371/journal.pone.0193667

**Published:** 2018-03-01

**Authors:** Camila A. Cotrim, Jose Sergio M. Soares, Bostjan Kobe, Marcelo Menossi

**Affiliations:** 1 School of Chemistry and Molecular Bioscience and Australia Infectious Diseases Research Centre, University of Queensland, Brisbane, Queensland, Australia; 2 Departamento de Genética, Evolução, Microbiologia e Imunologia, Instituto de Biologia, Universidade Estadual de Campinas, Campinas, São Paulo, Brazil; Russian Academy of Medical Sciences, RUSSIAN FEDERATION

## Abstract

UDP-glucose pyrophosphorylase (UGPase) is found in all organisms and catalyses the formation of UDP-glucose. In sugarcane, UDP-glucose is a branch-point in the carbon channelling into other carbohydrates, such as sucrose and cellulose, which are the major factors for sugarcane productivity. In most plants, UGPase has been described to be enzymatically active in the monomeric form, while in human and yeast, homo-octamers represent the active form of the protein. Here, we present the crystal structure of UGPase from sugarcane (ScUGPase-1) at resolution of 2.0 Å. The crystals of ScUGPase-1 reveal the presence of two molecules in the asymmetric unit and the multi-angle light scattering analysis shows that ScUGPase-1 forms a mixture of species ranging from monomers to larger oligomers in solution, suggesting similarities with the orthologs from yeast and human.

## Introduction

Sugarcane (*Saccharum ssp*. hybrids) is a highly productive C4 crop used for many centuries to produce sugar and, more recently, other value-added products such as ethanol and bioelectricity, through fermentation and burning of the sugarcane bagasse, respectively. Thereby, the accumulation of sucrose in the culm and the bagasse, cellulosic biomass, are the major yield components [1]. The particular importance of biochemical factors in the regulation of carbon (C)-partitioning to sucrose accumulation in the culm and cellulose synthesis are crucial to improve the sugarcane yield capacity. Among the various enzymes, UDP-glucose pyrophosphorylase (UGPase; EC 2.7.7.9) is important and essential in this carbon regulation, whereas the sugar, UDP-glucose, represents an important branch point in the C channel directing for synthesis of starch, sucrose or cellulose [2–4]. UGPase is an enzyme ubiquitously distributed in all types of organisms and catalyses the formation of UDP-glucose, the key precursor of the sucrose biosynthesis and cell wall metabolism in plants [5,6]. Moreover, UGPase is also involved in the biosynthesis of starch, converting UDP-glucose into ADP-glucose through the reaction coupled to ADP-glucose pyrophosphorylase (AGPase) activity [6]. UGPase also acts in concert with sucrose phosphate synthase (SPS) in the synthesis of sucrose in the source tissues [6], whereas its activity may be linked with the cellulose synthase complex in the formation of cellulose in the sink tissues [7].

To better comprehend the regulation and activity in the reactions involved in the saccharide metabolism, UGPases from different types of organisms have been characterized over the years. It is known that oligomerization plays a role in the regulatory process of these enzymes affecting their function and activity. For instance, UGPase from barley has been shown to exist as a mixture of monomers, dimers and higher oligomeric forms is solution, with the monomer being the most active form [8,9]. Similarly, UGPase from *Arabidopsis thaliana* also exists as a monomer in solution, although a dimer has been observed in the crystal structure [10]. On the other hand, the yeast and human orthologs have been described to form active octameric complexes [11,12]. Recently, the UGPase from sugarcane (ScUGPase-1) was characterized, showing that the enzymatic activity and regulatory mechanism are similar to those reported for other UGPases [13]. Moreover, small angle X-ray scattering (SAXS) data showed that ScUGPase-1 exists as a combination of monomers, dimers and higher oligomers in solution, with the monomeric envelope very similar to the momoner of the crystal structure of *A*. *thaliana* UGPase [13].

In this study, we present the crystal structure of ScUGPase-1 at 2.0 Å resolution. Structural analysis shows high structural similarity with other UGPase orthologs. Multi-angle light scattering (MALS) analysis shows a possible octamer of the recombinant protein in solution, consistent with the crystal structure described for the human and yeast orthologs.

## Material and methods

### Cloning, expression and purification of ScUGPase-1

The *ScUGPase-1* gene (KF278717) was obtained from the Brazilian SUCEST project database (http://www.sucest-fun.org/), with the Sugarcane Assembled Sequence number SCQGLR1062D04.g and cloned into the pENTR-D/TOPO vector, following cloning into the pET160- vector containing a hexa histidine-tag at the N-terminal, as described by [13]. The final construct was transformed into *E*. *coli* BL21 (DE3) strain for protein expression.

The expression was performed in 6 L LB medium containing 100 μg/mL ampicillin. Cells were grown at 37°C until OD_600_ of 0.6 was reached and protein expression was induced by addition of 1 mM isopropyl 1-thio-β-D-galactopyranoside (IPTG) for 4 h at 37°C. The cells were harvested by centrifugation (20 min, 5 000 *x* g) and resuspended in buffer A (50 mM Tris/HCl pH 8.0; 100 mM NaCl). The cells were lysated by sonication on ice and cell debris were removed by centrifugation (30 min, 20 000 x g). The supernatant was loaded onto a 5 mL HisTrap HP column (GE Healthcare) on an ÄKTA^TM^ system (GE Healthcare) for nickel-affinity chromatography. The column was washed with buffer A until the absorbance at 280 nm reached the baseline and the protein was eluted with buffer containing 0.5 M imidazole. The His-tagged ScUGPase-1 was buffer-exchanged into 20 mM Tris/HCl pH 8.0; 20 mM NaCl using a Superdex 200 16/60 gel filtration column (GE Healthcare) and analysed by SDS-PAGE.

### Crystallization

The monomeric form of ScUGPase-1 was crystallised by using the hanging-drop vapour-diffusion method. 2 μL of ScUGPase-1 protein solution (8 mg/mL) were mixed with 1 μL or 2 μL of reservoir solution containing 100 mM MES sodium salt pH 6.5, 200 mM (NH_4_)_2_SO_4_ and 23% (w/v) PEG 8000, producing plate-form crystals after two days at 20°C. Crystals were looped-out and soaked in a cryoprotectant solution containing crystallization buffer and ethylene glycol (25% (v/v) before flash-cooling.

### Data collection and processing

Data collection was carried out on beamline MX2 at the Australian Synchrotron (AS) in Melbourne at a wavelength of 0.9537 Å. The crystal diffracted to 2.0 Å resolution and the collected data were processed (indexing and integration) using XDS [14] and scaled in the Aimless (CCP4) program [15]. The crystals have the symmetry of the P1 space group. There are two molecules in the asymmetric unit. Data collection statistics are listed in Table 1. Diffraction images are available at The SBGrid Data Bank (doi: 10.15785/SBGRID/551).

**Table 1:** Data-processing and refinement statistics for ScUGPase-1.

**Data Collection**	
Radiation source	MX2 (AS*)
Wavelength (Å)	0.9537
Space group	P1
Cell dimensions	
a, b, c (Å)	61.35, 66.79, 74.78
Angles (°)	75.62, 79.00, 77.52
Resolution range (Å)	42.73–2.00 (2.04–2.00)
Rmerge^a^	0.123 (0.493)
Rmeas (within I+/I-)	0.173 (0.697)
Rmeas (all I+ & I-)	0.138 (0.488)
Mean I//σ(I)	5.6 (2.1)
Completeness (%)	97.5 (95)
Multiplicity	2.0 (1.9)
Molecules in ASU	2
Model used for MR	1Z90 (Chain A)
**Refinement**	
No. reflections (work/test)	73174 (2000)
R_work_^b^, R_free_^c^ (%)	19.30/22.90
No. atoms	
Protein	7065
Ligand/ion	52
Solvent	353
R.m.s deviations	
Bond lengths (Å)	0.007
Bond angles (°)	0.838
Ramachandran plot: (% in favoured/outlier regions)	98.65/0.0
MolProbity clashscore	2.44
PDB code	5WEG

* Australian Synchrotron

^**a**^ R_merge_ = Σhkl(Σi(|Ihkl,i−〈Ihkl〉|))/Σhkl,i〈Ihkl〉, where Ihkl,i is the intensity of an individual measurement of the reflection with Miller indices h, k and l, and 〈Ihkl〉 is the mean intensity of that reflection. Calculated for IN−3 σ(I).

^**b**^ R_work_ = Σhkl(||Fobs,hkl|−|Fcalc,hkl||)/|Fobs,hkl|,where |Fobs,hkl| and |Fcalc,hkl| are the observed and calculated structure factor amplitudes, respectively.

^**c**^ R_free_ is equivalent to Rcryst but calculated with reflections (5%) omitted from the refinement process.

### Structure solution and refinement

The structure was solved by molecular replacement using the sequence of UGPase from *Arabidopsis thaliana* (AtUGPase) (PDB code: 1Z90; chain A), which shares 83% sequence identity with ScUGPase-1, as the search model in *Phaser* without any modification [16]. The structure was firstly rebuilt through *AutoBuild wizard* (Phenix) [17] followed by manual building based on F_o_-F_c_ and 2F_o_-F_c_ difference maps using the *Coot* program [18]. Refinements were carried out using *phenix*.*refine* [19], including non-crystallographic symmetry (NCS) and TLS refinement, with chain A divided into three groups and chain B into seven groups. All structural figures were created using PyMOL (Schrödinger).

### Size-exclusion chromatography (SEC)-coupled multi-angle light scattering (MALS)

SEC-MALS was performed using a Superdex 200 increase 5/150 column (GE Healthcare) combined with a Dawn Heleos II 11-angle light scattering detector coupled with an Optilab TrEX refractive index detector (Wyatt Technology). The experiments were carried out at room temperature with a protein concentration of 2.0 mg/mL and a flow rate of 0.2 mL/min in 20 mM Tris/HCl pH 8.0; 20 mM NaCl buffer. Molecular mass calculations were performed using the Astra6.1 software (Wyatt Technology). Input of the refractive increment (dn/dc values) was set at 0.186 in the molecular mass calculations, based on the premise that dn/dc is constant for unmodified proteins [20]. The molecular mass was determined across the protein elution peak.

### Multiple sequence alignment

Multiple sequence alignment were carried out using the MUSCLE algorithm [21] and optimized in Jalview [22].

### Protein Data Bank accession code

Coordinates and structure factors have been deposited in the Protein Data Bank under accession code 5WEG.

## Results and discussion

### Overall structure of ScUGPase-1

The crystal structure of ScUGPase-1 was determined by molecular replacement using the AtUGPase [10] as the search model. The crystals have the symmetry of the triclinic space group P1 and diffracted to 2.0 Å resolution. The structure, with initial R-work of 29.80% and R-free of 32.7%, was built manually and improved based on Fo-Fc and 2Fo-Fc maps to give final R-work and R-free values of 18.94% and 22.74%, respectively. Data processing and refinement statistics are shown in Table 1.

**Fig 1:**
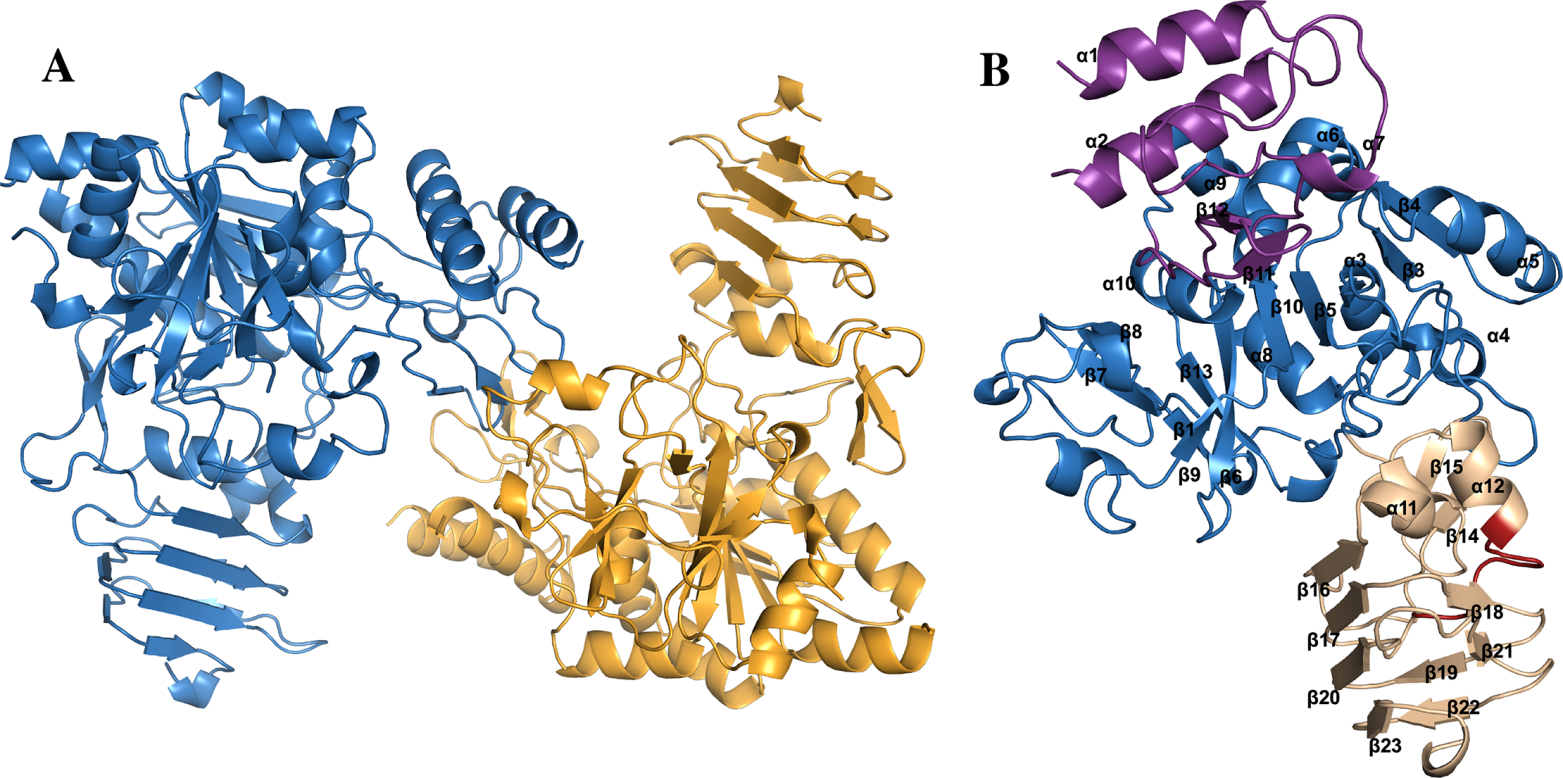
Crystal structure of ScUGPase-1. **(A)** Cartoon representation of the two molecules of ScUGPase-1 present in the asymmetric unit. Monomer A is coloured in blue and monomer B in orange. **(B)** The ScUGPase-1 monomer and its domains. The N-terminal domain is shown in purple, catalytic domain in blue and the C-terminal domain in wheat colour. The RFKS^419^IPSI motif is shown in red.

The crystals of ScUGPase-1 contain two molecules per asymmetric unit, labelled monomers A and B (Fig 1A), eight sulfate ions from the crystallization buffer, three molecules of ethylene glycol from the cryoprotectant solution and 353 water molecules. ScUGPase-1 was crystallized fused to an N-terminal His_6_-tag and TEV protease cleavage site, which added 32 residues to the chain (MHHHHHHGAGGCCPGCCGGGENLYFQGIITSL). Several surfaces loops could not be built due to poor electron density; these include residues A1-A12, A46-A49, A73-A75, A262-A263, B1-12, B47-B50, B72-B75 and B186-B189.

Similar to other UGPases [10–12,23,24], each monomer of ScUGPase-1 contains three domains: an N-terminal domain, a catalytic domain and a C-terminal domain (Fig 1B). The N-terminal domain consists of α1, α2, β11, β12, and three loops (Gln169-Gly190; Glu318-Pro328; Leu337-Ala342). The catalytic domain consists of a mixed nine-stranded β-sheet (β1-β6, β9, β10 and β13) as a core, surrounded by six α-helices (α3-α9), and resembles a Rossmann fold (Fig 1B). The C-terminal domain consists of 10 β-strands (β14-β23) and two α-helices (α11-α12), with the motif RFKS^419^IPSI, essential for the phosphorylation and binding with 14-3-3 protein [13,25], between α12 and β17.

### Structural comparison with AtUGPase

**Fig 2:**
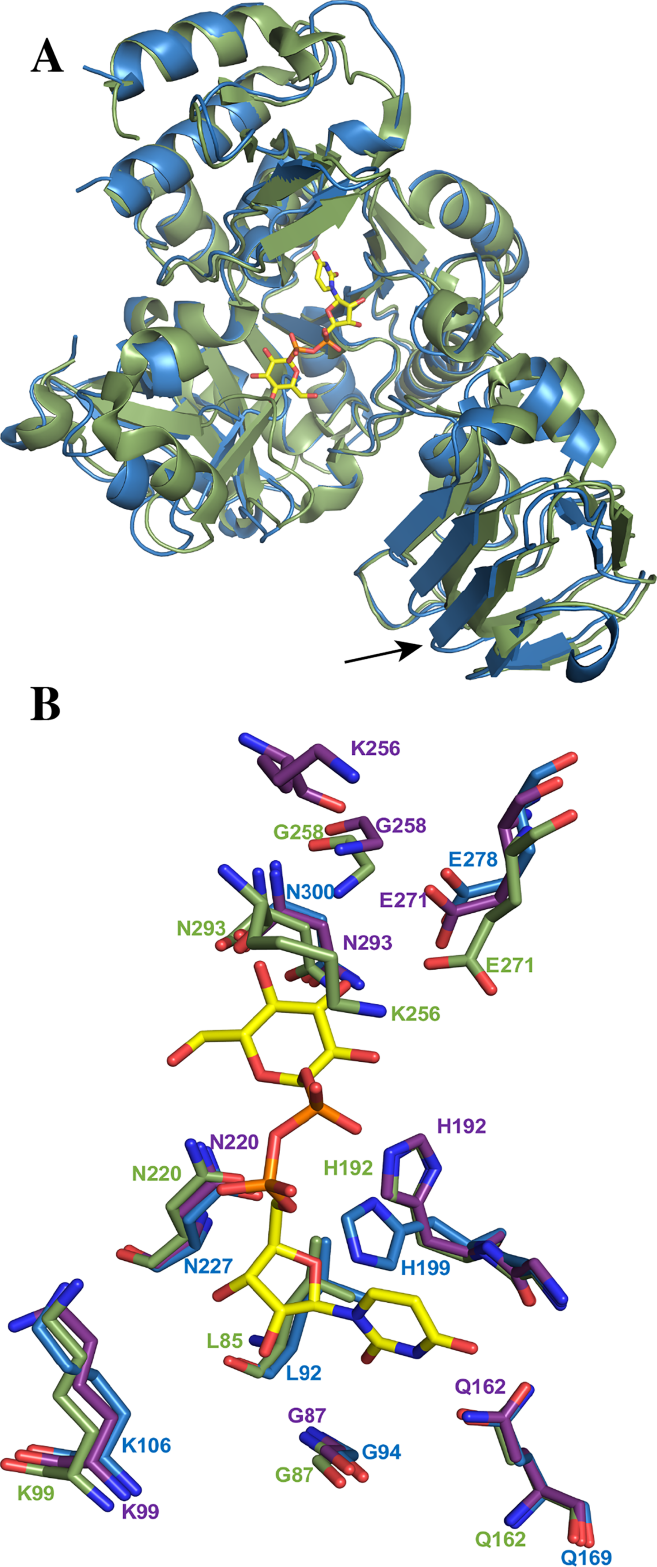
Structural comparison of ScUGPase-1 and AtUGPase. **(A)** Superposition of monomers of ScUGPase-1 (blue) and AtUGPase (green) (PDB code: 2ICY; RMSD value of 0.552 Å for 369 Cα atoms). UDP-glucose (yellow sticks) is shown in the active site. The arrow indicates β19 and β20 of ScUGPase-1; they are replaced by a unique β-strand in the AtUGPase structure. **(B)** Comparison of the active site of apo-ScUGPase-1 and AtUGPase containing UDP-glucose. Residues of AtUGPase involved in the stabilization of UDP-glucose are shown as green sticks and residues of ScUGPase-1 likely important for ligand-binding are shown in blue. The active site of the apo-AtUGPase (PDB code: 1Z90) is also included (purple sticks), showing the same arrangement as for AtUGPase containing UDP-glucose.

Among all the UGPase structures solved [10–12,23,24], ScUGPase-1 shares the highest sequence identity with UGPase from *Arabidopsis thaliana* (AtUGPase; 83%), and 54% and 56% with the human and yeast orthologs, respectively. Superposition of monomer A of ScUGPase-1 with the monomer of AtUGPase bound to UDP-glucose [10] (PDB code: 2ICY) shows a very low RMSD (root mean square deviation) value of 0.552 Å for 369 Cα atoms (Fig 2A). The ScUGPase-1 and AtUGPase structures differ in the C-terminal domain, whereas the β-strands β19 and β20 of ScUGPase-1 are replaced by a unique and longer β-strand in the AtUGPase structure (Fig 2A).

Analysis of the active site of AtUGPase shows that the UDP-glucose molecule is bound by several residues. The uridinyl group is coordinated by residues Gln162, Gly191 and Gly87 whereas the glucose portion of the molecule is coordinated by Asn220, Gly258, Glu271, Asn293 and Leu85. The β-phosphate is stabilized by His192 and Lys256 and the α-phosphate by Lys99 (Fig 2B).

**Fig 3:**
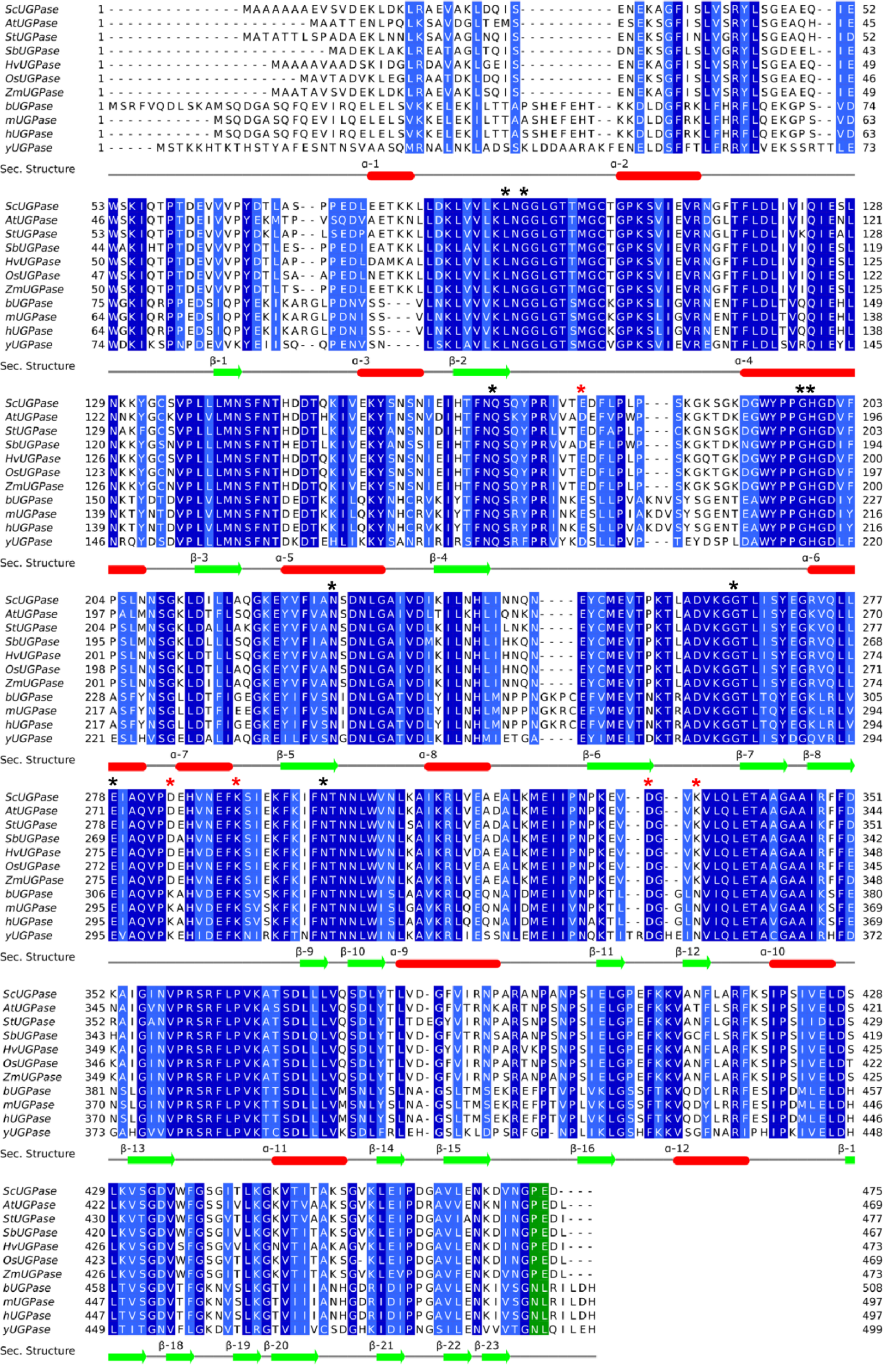
Multiple sequence alignment of UGPase orthologs. Proteins were aligned by MUSCLE [21] and the alignment optimized in Jalview [22]. The aligned sequences from top to bottom with their accession numbers are: ScUGPase-1 from *Saccharum* spp—sugarcane (A0A075E2Q1); AtUGPase from *Arabidopsis thaliana* (Q9M9P3); StUGPase from *Solanum tuberosum–*potato (P19595); SbUGPase from *Sorghum bicolor* (C5XSC5); HvUGPase from *Hordeum vulgare*–barley (Q43772); OsUGPase from *Oryza sativa–*rice (Q93X08); ZmUGPase from *Zea mays–*maize (B6T4R3); bUGPase from bovine–*Bos taurus* (Q07130); mUGPase from mouse–*Mus musculus* (Q91ZJ5-2); hUGPase from human–*Homo sapiens* (Q16851-2) and yUGPase from yeast–*Saccharomyces cerevisiae* (P32861). Region coloured in green corresponds to the residues in the C-terminal region involved in the end-to-end interactions in the yeast and human orthologs. Black asterisks (*) indicate the residues important for ligand binding, and red asterisks (*) indicate residues involved in the dimer interface in the crystal of ScUGPase-1. Elements of secondary structure are shown based on the crystal structure of ScUGPase-1. β-sheets and α-helices are shown in green and red, respectively.

Multiple sequence alignments show that all these residues are highly conserved in all UGPase orthologs analysed (Fig 3). Structural comparison of AtUGPase and ScUGPase-1 suggests that UDP-glucose likely binds to ScUGPase-1 active site in a similar way. Hence, the uridinyl group is likely stabilized by Gly94, Gln169 and Gly198, whereas the glucose portion would be coordinated by Asn227, Gly265, Glu278, Asn300 and Leu92, though no electron density was observed for Gly265 in the structure of ScUGPase-1.

A slight difference observed in the active site of ScUGPase-1 is the orientation of the side chain of His199. In the AtUGPase structure, the side chain is orientated closer to the β-phosphate, whereas in the ScUGPase-1 structure it is orientated closer to the α-phosphate. To evaluate whether this orientation could be caused by conformational changes induced by ligand, the structure of apo-AtUGPase (PDB code: 1Z90) was also analysed (S1A Fig). Structural comparison (Fig 2B) shows that the His192 assumes the same conformation in the apo-structure as in the UDP-glucose bound-structure. The side-chain likely has to rotate to allow substrate binding. Interestingly, in contrast to plant UGPases, which do not undergo large conformational changes (S1A Fig), *Leishmania major* (LmUGPase) and human UGPase (hUGPase) have been reported to undergo conformational changes induced by ligands [23,26,27]. Upon substrate binding, active monomers of LmUGPase suffers two processes that lead to large conformational modification. The first one is a significant relocation of the NB loop (nucleotide binding loop) towards the ligand, whereas the second one involves a movement of the SB loop (substrate binding loop) over the sugar moiety. These two conformational changes lead to a handle-like extension formed by β9, β10 and the connecting loop that adopt very different conformations in the apo- and ligand bond UGPase. They also demonstrated that the residues at the beginning and at the end of the handle, as well as the adjacent residues perform a 12° turn toward the sugar moiety in the catalytic domain (S1 Fig). Comparison of this region in the structure of ScUGPase shows that β7 and β8 (corresponding to β9 and β10 in LmUGPase) and the connecting loop are too short to undergo similar conformational change [23,26]. On the other hand, hUGPase is known to form active octamers that limit the conformational flexibility of subunits. To overcome this limitation, hUGPase stabilizes the ligand via an intermolecular mechanism named interlock, which involves R287 of one subunit and D456 of the neighbouring subunit [26]. These differences show that UGPases have diverse mechanisms to stabilize the substrate, depending of the organism.

#### Oligomeric state of ScUGPase-1

The oligomeric state of UGPase has been extensively studied, since oligomerization plays a regulatory role in the activity of this protein [9]. Studies have shown that other UGPases from plants exists as a mixture of monomers, dimers and higher oligomeric forms, with the monomer as the most active form [8,9]. *In vitro* analyses have also demonstrated that the oligomerization of UGPase is affected by buffer composition, with phosphate and Tris buffers promoting the appearance of several oligomers of different sizes, while MOPS and HEPES lead to UGPase de-oligomerization [9].

**Fig 4:**
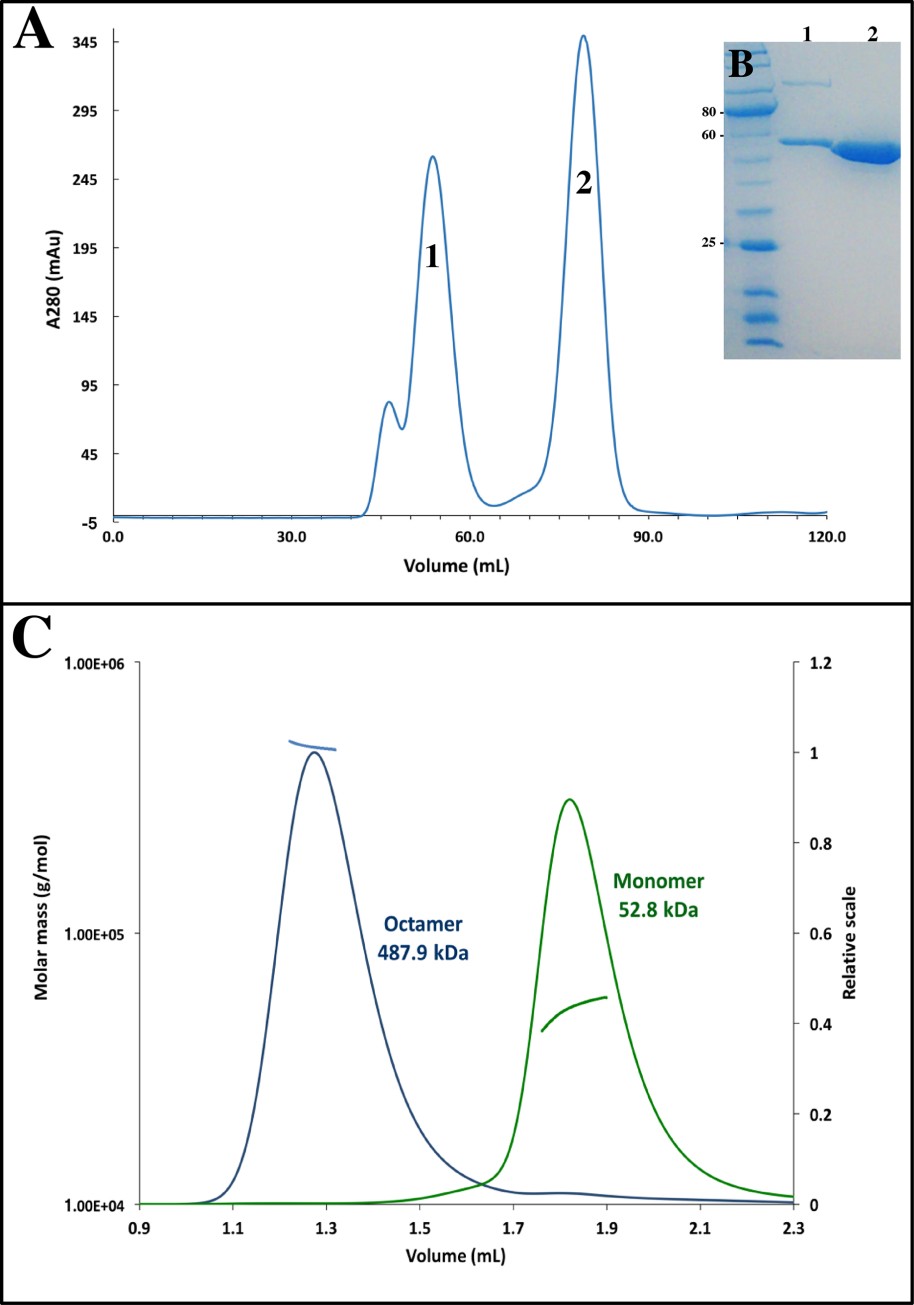
Size exclusion chromatography and MALS analysis. **(A)** Size exclusion chromatogram of ScUGPase-1. Peaks between 40 and 60 mL correspond presumably to higher oligomeric forms of ScUGPase-1, whereas the peak at 75 mL corresponds to the monomeric form. **(B)** Coomassie-stained gel under reducing conditions showing the purity of ScUGPase-1. **(C)** SEC-MALS analysis of ScUGPase-1. Blue and green lines indicate the trace from the refractive index detector during SEC for the octamer and monomer, respectively. Black lines on each peak correspond to the averaged molecular weight (Mw; y axis) distribution across the peak.

Size-exclusion chromatography conducted during the purification of ScUGPase-1, in the presence of Tris buffer, showed the presence of two main peaks (Fig 4A), indicating the existence of ScUGPase-1 as a mixture of monomers and higher oligomeric forms in solution. The crystal structure revealed the presence of two molecules per asymmetric unit, suggesting a possible dimer (Fig 2A). A putative dimer formation was also observed in the structure of AtUGPase [10], but with a different arrangement (S2 Fig).

Analysis of the possible dimer of ScUGPase-1 found in the crystal shows that the putative dimer interface is small and stabilized by hydrogen bonds between Glu178 (monomer A) and Lys291 (monomer B); Lys335 (monomer A) and Asp284 (monomer B); and between Asp332 (monomer A) and Lys335 (monomer B). These residues are conserved in all analysed UGPase orthologs from plants (Fig 3, shown with red asterisk), however, except for Asp284, none of these residues are relevant for the putative dimer interaction observed for AtUGPase. The “Protein interface and assemblies” (PISA) server [28] has been used to access potential interfaces relevant for protein-protein interactions. It has been reported that a minimum contact area of ~ 600 Å^2^ is required for protein-protein complexes. Analysis of ScUGPase-1 revealed a buried surface area of 480 Å^2^ between monomers A and B, and a complexation significance score (CSS) of 0, indicating the corresponding dimer is unlikely to be stable. A small buried surface area of 600 Å^2^ was also observed for the putative dimer of AtUGPase [10].

In order to get further insights into the oligomerization properties of ScUGPase-1, we performed multi-angle light scattering (MALS) using the samples corresponding to the monomer and the larger oligomeric form peaks (Fig 4A), at concentration of 2.0 mg/mL. Analysis of the monomer peak showed a molecular mass of 52.8 kDa (± 0.9%) (Fig 4B), close to the expected mass of 56.1 kDa. On the other hand, analysis of the peak corresponding to the higher oligomeric form showed a molecular mass of 487.9 kDa (± 0.7%) (Fig 4B), which would be consistent with an octamer (theoretical mass 448.8 kDa). Octamers have been found in solution and in the crystal structure of the yeast (yUGPase) and human (hUGPase) orthologs [11,12]. The crystal structure of both proteins revealed that the C-terminal domain is essential for the dimer formation through an end-to-end arrangement, and four dimers assemble into an octamer, the fully active form of the protein (S3A and S3B Fig) [11,12]. The RMSD values between the monomer structures of ScUGPase-1 and hUGPase and yUGPase are 1.14 Å (376 Cα atoms) and 0.804 Å (399 Cα atoms), respectively, showing a high structural similarity (S4 Fig).

We could not identify in the crystal structure ScUGPase-1 any interaction among the symmetry-related molecules that are equivalent to the interactions between human and yeast molecules in the octamers. Interestingly, using small angle x-ray scattering (SAXS) analysis, Soares et al., 2014 have shown that at low concentrations (0.64 mg/mL) oxidized ScUGPase-1 exists as dimer in solution and that the dimer envelope observed suggests a dimer interface via C-terminal domain in an arrangement very similar to yUGPase and hUGPase. Yu & Zheng 2012 have reported that in humans and possibly in other higher eukaryotes, Asn491 and Leu492 are essential for dimer formation. Multiple sequence alignment shows that indeed these two residues are conserved in human, yeast, bovine and mouse proteins, but not in plant proteins, which have a proline and glutamic acid instead (Fig 3, green). Thereby, the residues and the mechanism involved in the putative end-to-end interactions in ScUGPase-1 are still unknown, but unlikely to be the same as observed in the structures from human and yeast. The role of the C-terminal domain in the oligomerization of UGPase has been investigated in barley, which shares a sequence identity of 93% with ScUGPase-1. In studies using truncated proteins, it has been demonstrated that deletion of the last eight residues of the C-terminal region not only increases the UGPase activity, but also keeps the protein mostly as a monomer, suggesting that this region may play a role in the oligomerization of UGPase protein [29].

Altogether, our results suggest the existence of ScUGPase-1 as a mixture of monomers, dimers and octamers in solution, which might adopt a similar structural arrangement as described for human and yeast orthologs. Further studies are necessary in order to get more insights about the oligomeric structure of ScUGPase-1.

## Conclusions

In this study, we present the first crystal structure of UGPase-1 from sugarcane at a resolution of 2.0 Å. Structural comparisons to the UGPase from *Arabidopsis thaliana* (AtUGPase) reveals a high structural similarity, providing insights into the active site of ScUGPase-1. Multi-angle light scattering (MALS) results indicate the presence of a possible octamer in solution, which might be formed by four dimers through end-to-end arrangements of two monomers similar to yeast and human proteins.

## Supporting information

S1 Fig Structural comparison of apo- and ligand-bound UGPase. **(A)** Surface representation of apo-AtUGPase (green) (PDB code: 1Z90) and AtUGPase bound to UDP-glucose (purple) (PDB code: 2ICX). RMSD value of 0.576 Å for 373 Cα atoms indicates small conformational changes induced by ligand. **(B)** Surface representation of LmUGPase comparing the open (brown) (PDB code: 2OEF) and closed conformation (light green) (PDB code: 2OEG). RMSD of 1.826 Å for 373 Cα atoms.
(TIF)

S2 Fig Structural comparison of ScUGPase-1 and AtUGPase. Superposition of the ScUGPase-1 putative dimer (blue and orange) with the dimer of AtUGPase (green and grey) (PDB code: 2ICX). RMSD value of 20.88 Å for 875 Cα atoms.
(TIF)

S3 Fig Structure of human UGPase. **(A)** Diagram showing the dimer formation through an end-to-end arrangement in the C-terminal. **(B)** Top and side view of the hUGPase octamer. The four dimers are shown in red, cyan, green and yellow. (PDB code: 3R2W).
(TIF)

S4 Fig Structural comparison of ScUGPase-1 with other orthologs. **(A)** Superposition of ScUGPase-1 (blue) and hUGPase (red) (PDB code 3RW2) shows RMSD value of 1.14 Å over 376 Cα atoms. **(B)** Superposition of ScUGPase-1 (blue) with yUGPase (pink) (PDB code: 2I5K). RMSD value of 0.804 Å over 399 Cα atoms.
(TIF)
