# Supplementary figures and images for: Crystal structure and insights into the oligomeric state of UDP-glucose pyrophosphorylase from sugarcane
Source: PLoS One. 2018 Mar 1;13(3):e0193667. doi: 10.1371/journal.pone.0193667 (PMC5832301; doi:10.1371/journal.pone.0193667)

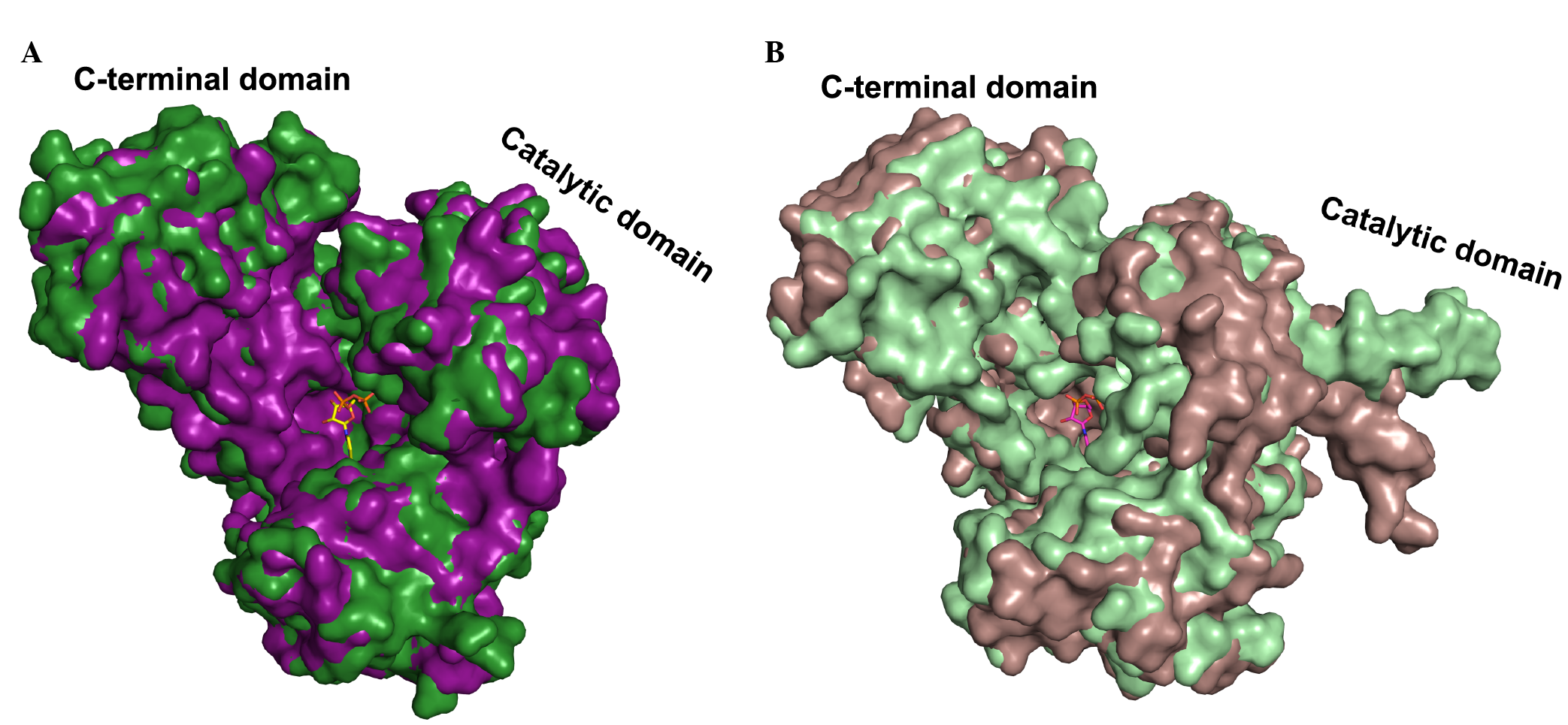

Supplement: S1 Fig — (A) Surface representation of apo-AtUGPase (green) (PDB code: 1Z90) and AtUGPase bound to UDP-glucose (purple) (PDB code: 2ICX). RMSD value of 0.576 Å for 373 Cα atoms indicates small conformational changes induced by ligand. (B) Surface representation of LmUGPase comparing the open (brown) (PDB code: 2OEF) and closed conformation (light green) (PDB code: 2OEG). RMSD of 1.826 Å for 373 Cα atoms. (TIF) [file pone.0193667.s001.tif]

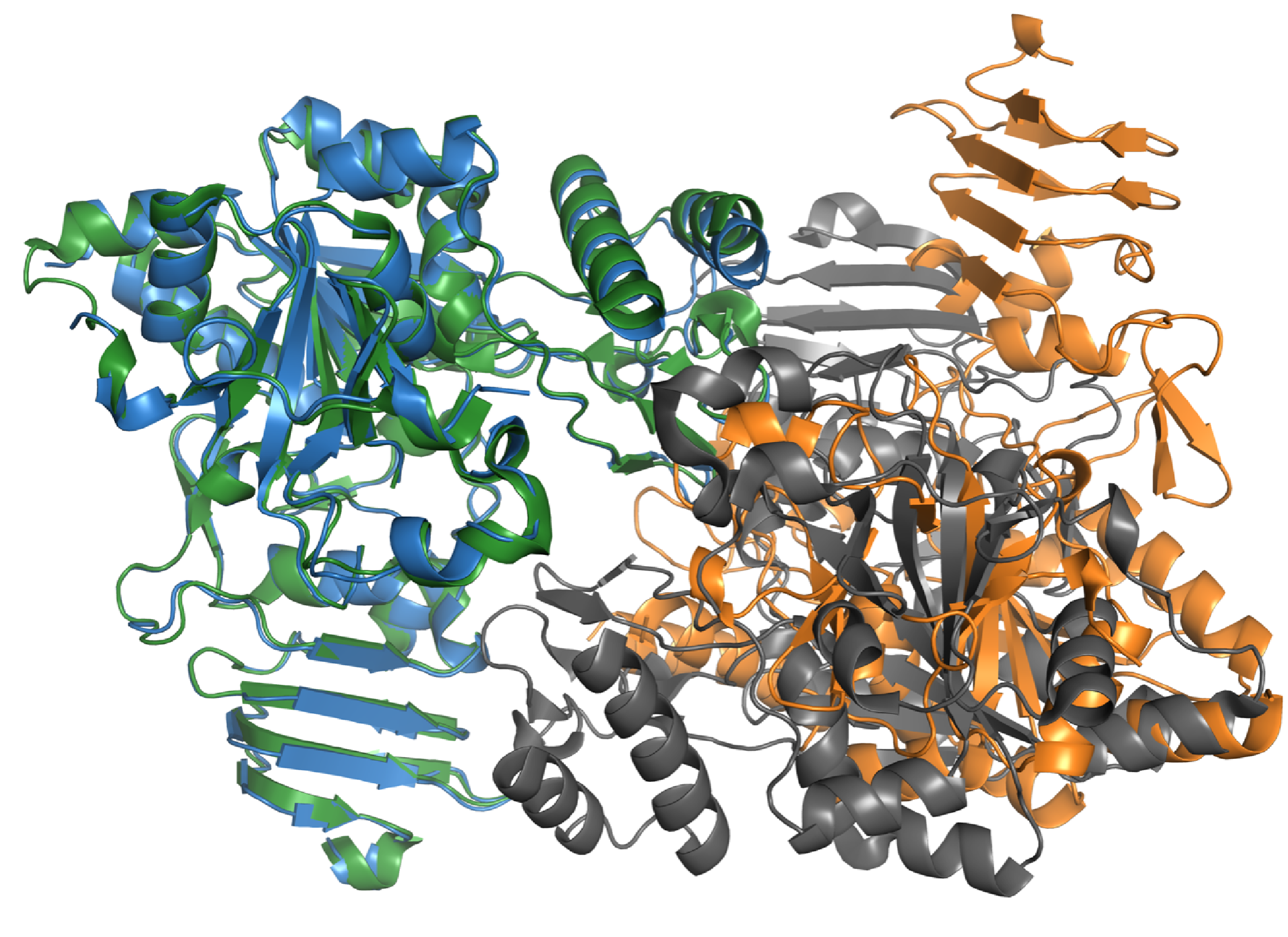

Supplement: S2 Fig — Superposition of the ScUGPase-1 putative dimer (blue and orange) with the dimer of AtUGPase (green and grey) (PDB code: 2ICX). RMSD value of 20.88 Å for 875 Cα atoms. (TIF) [file pone.0193667.s002.tif]

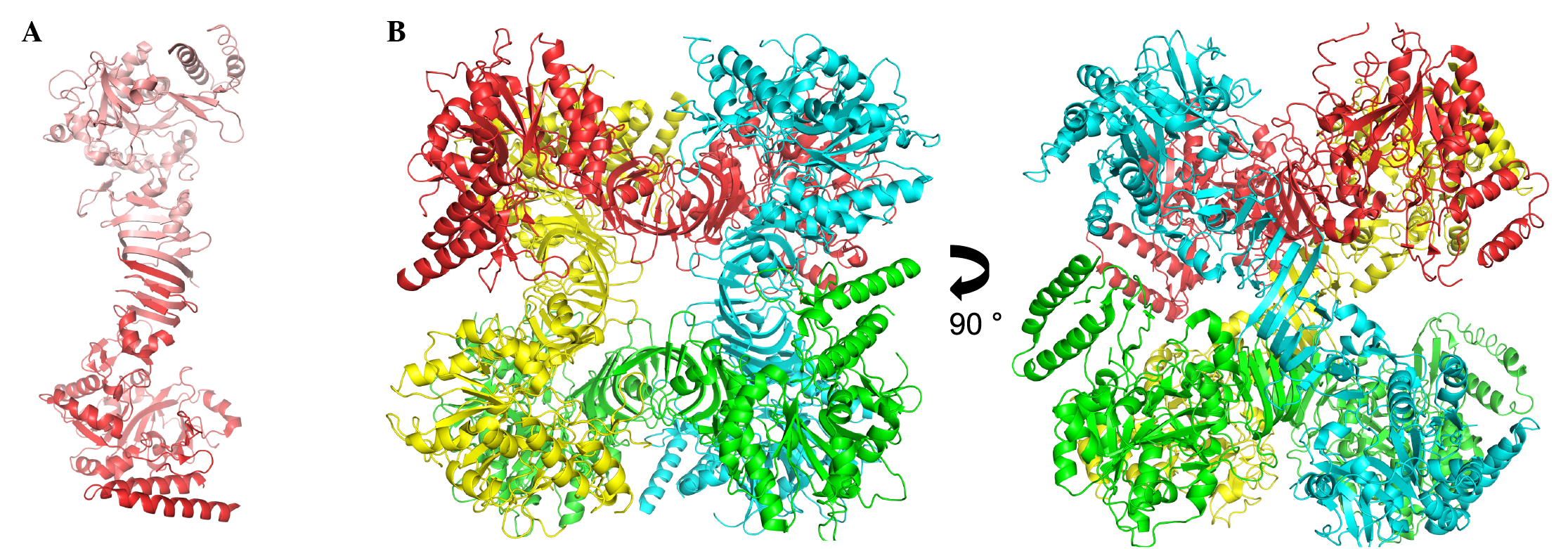

Supplement: S3 Fig — (A) Diagram showing the dimer formation through an end-to-end arrangement in the C-terminal. (B) Top and side view of the hUGPase octamer. The four dimers are shown in red, cyan, green and yellow. (PDB code: 3R2W). (TIF) [file pone.0193667.s003.tif]

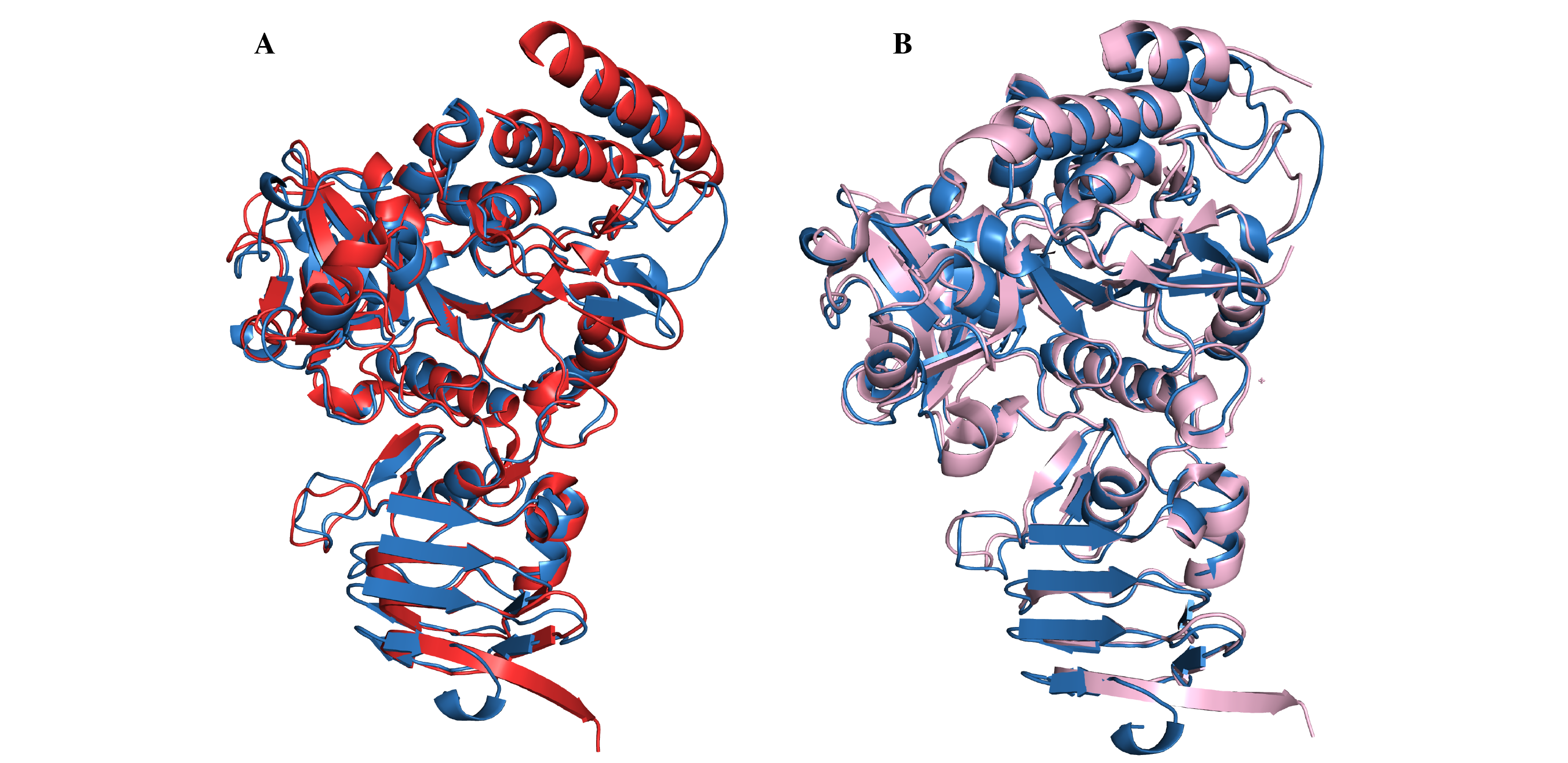

Supplement: S4 Fig — (A) Superposition of ScUGPase-1 (blue) and hUGPase (red) (PDB code 3RW2) shows RMSD value of 1.14 Å over 376 Cα atoms. (B) Superposition of ScUGPase-1 (blue) with yUGPase (pink) (PDB code: 2I5K). RMSD value of 0.804 Å over 399 Cα atoms. (TIF) [file pone.0193667.s004.tif]
